# Incidence and Geographic Distribution of Extensively Drug-Resistant Tuberculosis in KwaZulu-Natal Province, South Africa

**DOI:** 10.1371/journal.pone.0132076

**Published:** 2015-07-06

**Authors:** Jennifer R. Lim, Neel R. Gandhi, Thuli Mthiyane, Koleka Mlisana, Julie Moodley, Prenika Jaglal, Neeshan Ramdin, James C. M. Brust, Nazir Ismail, Roxana Rustomjee, N. Sarita Shah

**Affiliations:** 1 Albert Einstein College of Medicine and Montefiore Medical Center, Bronx, New York, United States of America; 2 Emory University Rollins School of Public Health, Atlanta, Georgia, United States of America; 3 University of KwaZulu-Natal, Durban, South Africa; 4 National Health Laboratory Service, Durban, South Africa; 5 Centre for Tuberculosis, National Institute for Communicable Diseases, National Health Laboratory Service, Johannesburg, South Africa; 6 Department of Medical Microbiology, University of Pretoria, Pretoria, South Africa; 7 Medical Research Council, Cape Town, South Africa; Institut de Génétique et Microbiologie, FRANCE

## Abstract

South Africa is experiencing a widespread drug-resistant tuberculosis epidemic, although data are limited regarding the current situation. This study finds that the extensively drug-resistant tuberculosis (XDR-TB) incidence in KwaZulu-Natal increased to 3.5 cases/100,000 (776 cases) in 2011-2012. XDR-TB cases are widely distributed geographically, with the majority of districts experiencing a rise in incidence.

## Introduction

Extensively drug-resistant tuberculosis (XDR-TB) is a growing public health threat and has been identified from 92 countries, to date [1,2]. XDR-TB significantly limits treatment options, resulting in prolonged infectious periods, ongoing transmission and clinical decline, and mortality rates of 42–98% in high HIV prevalence settings [3–5]. South Africa (SA) has the highest reported prevalence of drug-resistant TB in sub-Saharan Africa [1], with XDR-TB first identified in a rural area of KwaZulu-Natal province in 2005 [4]. Although initially considered to be an isolated “outbreak,” subsequent epidemiologic data confirmed that the XDR-TB epidemic was widespread in KwaZulu-Natal province and throughout SA [5–8].

Since the initial reports of XDR-TB, there have been notable investments in combatting the epidemic in SA. Increased political commitment and funding has resulted in a multi-sectored response among stakeholders. Community education and mobilization activities have increased awareness among patients and providers to seek early testing and referral for treatment, as well as prevent nosocomial transmission. Decentralized care for drug-resistant TB to regional and district hospitals has facilitated earlier treatment with second-line drugs. Provincial and national TB reference laboratories have improved quality assurance of diagnostic testing and strengthened surveillance systems [9–11]. These interventions are likely to have impacted the epidemiology of XDR-TB in KwaZulu-Natal province since the start of the epidemic.

Evidence from successful national TB control programs in Estonia, Latvia, Hong Kong, Singapore, and the United States have demonstrated declines in drug-resistant TB incidence, though the reversal of these epidemics was gradual even with intensive interventions [12]. There has been no province-wide assessment of XDR-TB incidence in SA since 2007. We sought to describe changes in incidence and geographic distribution of XDR-TB in the KwaZulu-Natal province since 2007, in order to highlight priority districts and inform provincial TB control efforts.

## Methods

KwaZulu-Natal is the second most populous province in SA, with 10.3 million people. It is divided into 11 health districts with 74 provincial hospitals and 667 provincial clinics and community health centers. The provincial TB incidence is 1,156 per 100,000 and the HIV prevalence is 15.8% among those above 2 years of age [13].

This cross-sectional study analyzed culture and drug-susceptibility testing (DST) records of the provincial TB referral laboratory in KwaZulu-Natal. All provincial TB cultures were performed at this laboratory as part of routine medical care. South African national guidelines recommend DST for TB suspects who have defaulted or failed treatment, TB patients who have repeatedly positive smear microscopy results, and people at high risk of drug-resistant TB, including MDR and XDR-TB contacts, health care personnel and prisoners.

TB cultures are performed using the BACTEC Mycobacteria Growth Indicator Tube (MGIT) 960 system. MGIT-960 positive cultures undergo line probe assay (LPA), [GenoType MTBDRplus assay, Hain Lifescience, Nehren, Germany]; any LPA-resistant samples undergo DST using the 1% proportion method on Middlebrook 7H10 agar for the following drugs: isoniazid, rifampin, streptomycin, ofloxacin, and kanamycin. We included all sputum specimens with culture-confirmed XDR-TB collected between October 2010 and December 2012. Patients with multiple XDR-TB specimens were regarded as a single case with the earliest date of specimen collection selected.

To describe the geographic distribution of XDR-TB, government health facilities from which an XDR-TB sputum sample was sent were grouped by district. We calculated simple frequencies to determine the proportion of cases identified by each district and incidence rate per 100,000 population. Provincial census data from 2011 was used to obtain the population for each district [14]. Percent change was calculated to compare incidence rates to those of 2007 [6].

The study protocol was approved by the Institutional Review Boards (IRB) at Emory University (#00060394), Albert Einstein College of Medicine, University of KwaZulu-Natal and the KwaZulu-Natal Department of Health. As these samples were collected for routine clinical care, patients were not asked to provide informed consent at the time of the clinical encounters. For the intentions of this study, there was no requirement for informed consent since all data used were previously collected during the course of routine medical care and did not pose any additional risks to the patients. Patient information was anonymized and de-identified for analysis.

## Results

Between October 2010 and December 2012, a total of 776 XDR-TB cases were identified in KwaZulu-Natal province, equating to a province-wide incidence of 3.5 XDR-TB cases per 100,000 population. Women represented over half (59%, 329 of 555 with available data) of the cases. The median age was 33 years (range: 0–76 years; data available for n = 616). Although the *incidence varied by district, nearly all districts had an incidence of greater than 1.0 XDR-TB cases per 100,000 population, with the highest incidences in Umzinyathi (12.1 cases/100,000), Ethekwini (4.3 cases/100,000), and uMgungundlovu (3.4 cases/100,000) (Table 1).

**Table 1:** Cases of XDR-TB in KwaZulu-Natal province, South Africa, October 2010-December 2012.

District	XDR cases No. (%)	Population*(2011)	XDR cases/ 100,000 population (study period)	XDR cases/ 100,000 population (2007)^†^	Change in Incidence(%)
Total	776	10,267,301	3.5	3.1	+13%
Umzinyathi	134 (17.3)	510,838	12.1	34.1	-64%
Ethekwini	322 (41.5)	3,442,361	4.3	1.9	+127%
uMgungundlovu	75 (9.7)	1,017,763	3.4	4.0	-15%
Ugu	52 (6.7)	722,484	3.3	0.9	+269%
Zululand	50 (6.4)	803,575	2.9	0.3	+857%
Uthungulu	53 (6.8)	907,519	2.7	1.0	+170%
Umkhanyakude	28 (3.6)	625,846	2.1	0.3	+588%
uThukela	23 (3.0)	668,848	1.6	0.8	+98%
iLembe	19 (2.4)	606,809	1.1	1.0	+9%
Amajuba	11 (1.4)	499,839	1.0	0.3	+239%
Sisonke	9 (1.2)	461,419	0.9	2.0	-55%

*Statistics Soth Africa, 2012. [14]

^†^Moodley et al. PLOS ONE 011. [6]

**Fig 1:**
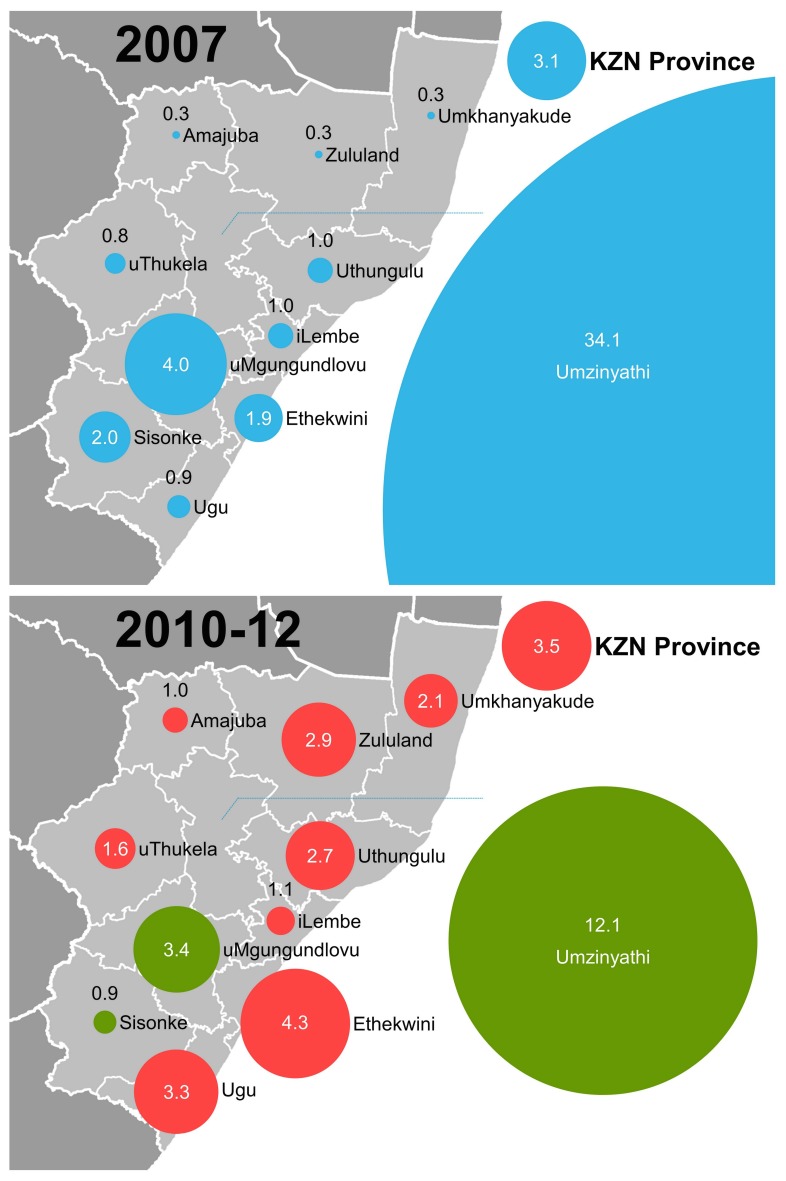
Change in incidence of XDR-TB in KwaZulu-Natal province, South Africa, 2007 to 2010–12. Panel A shows the XDR-TB incidence (per 100,000 population) in each district in 2007. Panel B shows the average XDR-TB incidence by district in 2010–2012. The size of the circles is proportional to the incidence rate. The colors in panel B represents the change in incidence compared to 2007 data: red, increase; green, decrease.

The average number of XDR-TB cases in the province each year increased from 270 to 358 and the overall incidenc e increased by 13%, compared to 2007 (Table 1). XDR-TB incidence increased in 8 of 11 districts, with the magnitude of increases ranging from 9% to 857% (Fig 1). In Umzinyathi and uMgungundlovu, although the incidence declined over this time period, it remained among the highest in the province at 12.1 and 3.4 XDR-TB cases/100,000, respectively. The burden of XDR-TB cases remains concentrated in Umzinyathi eThekwini, and uMgungundlovu; eThekwini and uMgungundlovu are home to Durban and Pietermaritzburg, the two largest cities in KwaZulu-Natal that account for nearly half of the provincial population.

## Conclusions

South Africa remains in the top 10 countries with the highest burden of drug-resistant TB worldwide [15]. Our study showed that XDR-TB incidence has increased in KwaZulu-Natal province, South Africa, and exceeds incidence rates of all TB in certain low-incidence countries, such as the United States. The XDR-TB epidemic is widely distributed throughout the province, with most districts experiencing a several-fold increase in incidence since 2007. These findings provide important epidemiologic information about the evolution of this epidemic and targeting of public health efforts.

A surprising finding from this study is that investments in drug-resistant TB control have not resulted in reduced case rates in most districts. Interestingly, the most dramatic increases have been in the rural districts, suggesting improved access to health care services could be an important reason for the increase. Improved patient and healthcare provider education may have paradoxically led to increased case detection resulting in higher numbers for the current study compared with a false low baseline in 2007. In addition, use of the Hain line probe assay (LPA) since 2009, followed by introduction of GeneXpert in 2011 may have contributed to increased testing and case detection province-wide. Further analysis of the molecular epidemiology of XDR-TB may be a more sensitive indicator of the impact of control measures, namely infection control interventions to prevent nosocomial transmission, and earlier diagnosis and access to treatment to reduce the infectious period. Continued investments in prevention, care and treatment are needed to sustain progress. Initial resource allocation may be costly, but are cost-effective in maximizing health benefits [16]. This is further evident with the largest decline observed in the Umzinyati district, the epicenter of the Tugela Ferry outbreak that received the earliest and most attention. Thus, despite the increases in incidence in this study for most of the other districts, downward trends are expected over a greater period of time as control measures extend their reach across the widespread epidemic in KwaZulu-Natal.

HIV infection has been a major driving force on the escalating burden of TB throughout sub-Saharan Africa. In KwaZulu-Natal province, 65% of incident TB cases and over 80% of drug-resistant TB cases are co-infected with HIV [14,17]. Antiretroviral therapy (ART) has been shown to improve survival among XDR-TB patients [3]. Prolonged duration of ART has limited impact on TB incidence among HIV-infected patients [18]. It is critical to address complex disease management issues, such as drug-drug interactions, treatment monitoring, and adherence to ART and second-line and third-line anti-TB treatment in order to achieve improved outcomes. Additionally, since TB disease in HIV co-infected populations is driven by a high background incidence of TB, it is important to address TB control in HIV-negative populations as well.

A major strength of this study is the centralized laboratory for culture and drug-susceptibility testing (DST) of TB patients in KwaZulu-Natal at the time of this study that allowed for complete capture of all XDR-TB diagnoses in the province. A limitation, however, is variation in provider adherence to TB culture and DST guidelines, which may introduce ascertainment bias into our study. If providers were more likely to have requested culture and DST in 2011–2012 than previously, it may account for the rise in incidence observed in many districts. Data were not available to examine changes in culture-taking practices since 2007. It should also be noted that the target group for DST are high-risk patients for drug resistance; thus, the rates may be over-represented. However, the same DST policy was in place in 2007 and the validity of the trend would remain true. Patients who attended private health facilities were not included in this analysis; the role of private laboratories in diagnosing XDR-TB is very limited, and literature describing these data was not available. Lastly, district-level data for MDR-TB was not readily available for the study timeframe to allow for comparisons of changes in MDR-TB vs. XDR-TB case rates. However, MDR-TB case counts for KwaZulu-Natal province have remained relatively stable from 2005 through 2010, the latest year for which reliable data are available [19].

KwaZulu-Natal province has the highest prevalence of XDR-TB within South Africa and has implemented multiple programmatic initiatives to combat this complex epidemic. XDR-TB rates in other provinces may differ, in part due to differing HIV prevalence, socioeconomic factors, access to MDR-TB treatment and diagnostic testing capability. The last national TB drug resistance survey was conducted in 2001–2002, but specimens were only tested for first-line drugs and data on XDR-TB were unavailable [20]. A drug resistance survey is currently underway in all nine provinces that will inform the national TB program on setting evidence-based priorities for combating MDR and XDR-TB in SA [21].

## References

[1] World Health Organization. (2013) Global Tuberculosis Report 2013. Geneva: The Organization; 2013. Report No.: Publication no. WHO/HTM/TB/2013.11.

[2] Shah N, Centers for Disease Control and Prevention. (2006) Emergence of Mycobacterium tuberculosis and Extensive Resistant to Second-Line Drugs—Worldwide, 2000–2004. Morbidity and Mortality Weekly Report 55:301–5. 16557213

[3] O'Donnell MR, Padayatchi N, Kvasnovsky C, Werner L, Master I, Horsburgh CR Jr. (2013) Treatment outcomes for extensively drug-resistant tuberculosis and HIV co-infection. Emerging infectious diseases. 19(3):416–24. 10.3201/eid1903.120998 23622055 PMC3647656

[4] Gandhi NR, Moll A, Sturm AW, Pawinski R, Govender T, Lalloo U, et al (2006) Extensively drug-resistant tuberculosis as a cause of death in patients co-infected with tuberculosis and HIV in a rural area of South Africa. Lancet. 368(9547):1575–80. 17084757 10.1016/S0140-6736(06)69573-1

[5] Pietersen E, Ignatius E, Streicher EM, Mastrapa B, Padanilam X, Pooran A, et al (2014) Long-term outcomes of patients with extensively drug-resistant tuberculosis in South Africa: a cohort study. Lancet. 383(9924):1230–9. 10.1016/S0140-6736(13)62675-6 24439237

[6] Moodley P, Shah NS, Tayob N, Connolly C, Zetola N, Gandhi N, et al (2011) Spread of extensively drug-resistant tuberculosis in KwaZulu-Natal province, South Africa. PloS one. 6(5):e17513 10.1371/journal.pone.0017513 21655324 PMC3104985

[7] Wallengren K, Scano F, Nunn P, Margot B, Buthelezi SS, Williams B, et al (2011) Drug-Resistant tuberculosis, KwaZulu-Natal, South Africa, 2001–2007. Emerging infectious diseases. 17(10):1913–6. 10.3201/eid1710.100952 22000370 PMC3310642

[8] Chihota VN, Muller B, Mlambo CK, Pillay M, Tait M, Streicher EM, et al (2012) Population structure of multi- and extensively drug-resistant Mycobacterium tuberculosis strains in South Africa. Journal of clinical microbiology. 50(3):995–1002. 10.1128/JCM.05832-11 22170931 PMC3295122

[9] National Institute for Communicable Diseases DotNHLS, Republic of South Africa. (2009) Annual Report 2009.

[10] Abdool Karim SS, Churchyard GJ, Karim QA, Lawn SD. (2009) HIV infection and tuberculosis in South Africa: an urgent need to escalate the public health response. Lancet. 374(9693):921–33. 10.1016/S0140-6736(09)60916-8 19709731 PMC2803032

[11] Department of Health RoSA. (2007) Tuberculosis Strategic Plan for South Africa, 2007–2011.

[12] Christopher D. (2009) Doomsday postponed? Preventing and reversing epidemics of drug-resistant tuberculosis. Nature Reviews Microbiology. 7(1):81–7. 10.1038/nrmicro2048 19079354

[13] Office of the Premier. (2012) Multi-Sectoral Provincial Strategic Plan for HIV and AIDS, STI and TB 2012–2016 for KwaZulu-Natal. Pietermaritzburg, KwaZulu-Natal, South Africa.

[14] Statistics South Africa RoSA. Census 2011 Municipal report—KwaZulu-Natal 2012. Report No.: 03-01-53.

[15] World Health Organization. (2014) Global Tuberculosis Report 2014 Supplement: Drug-Resistant TB Surveillance & Response. Geneva: The Organization; 2014. Report No.: Publication no. WHO/HQ/TB/2014.12.

[16] Frieden TR, Fujiwara PI, Washko RM, Hamburg MA. (1995) Tuberculosis in New York City—turning the tide. The New England journal of medicine. 333(4):229–33. 7791840 10.1056/NEJM199507273330406

[17] Gandhi NR, Shah NS, Andrews JR, Vella V, Moll AP, Scott M, et al (2010) HIV Coinfection in Multidrug- and Extensively Drug-Resistant Tuberculosis Results in High Early Mortality. American Journal of Respiratory and Critical Care Medicine. 181(1):80–6. 10.1164/rccm.200907-0989OC 19833824

[18] Gupta A, Wood R, Kaplan R, Bekker LG, Lawn SD. (2012) Tuberculosis incidence rates during 8 years of follow-up of an antiretroviral treatment cohort in South Africa: comparison with rates in the community. PloS one. 7(3):e34156 10.1371/journal.pone.0034156 22479548 PMC3316623

[19] Ndjeka N. Department of Health RoSA. Multi-Drug Resistant Tuberculosis: Strategic Overview on MDR-TB Care in South Africa. Medicines San Frontieres. 5 Mar 2014. Available: https://www.msf.org.za/sites/msf.org.za/files/Publications/Strategic_overview_of_MDR_TB_RSA.pdf. Accessed 8 May 2015.

[20] Weyer K, Lancaster J, Brand J, van der Walt M, Levin J. (2004) Survey of Tuberculosis Drug Resistance in South Africa 2001–2002: Final Report. Pretoria: Medical Research Council.

[21] Ihekweazu C, Ismail N, Koornhof H. National Institute for Communicable Diseases. (2012) Anti-Tuberculosis Drug Resistance Survey for South Africa, 2012–2013. Communicable Diseases Surveillance Bulletin. 10(4):79–81.

